# Supplementary material for: The comparative osteology of Plesiochelys bigleri n. sp., a new coastal marine turtle from the Late Jurassic of Porrentruy (Switzerland)
Source: PeerJ. 2017 Jun 28;5:e3482. doi: 10.7717/peerj.3482 (PMC5493033; doi:10.7717/peerj.3482)
Supplement: Supplemental Information 3 — Thickness was measured on the left and right side, respectively, and approximately at the middle of each neural. Measurements are expressed in millimeters. All specimens are housed at the MJSN. [file peerj-05-3482-s003.docx]

***Plesiochelys bigleri***

| **Specimen** | **Neural number** | **Length** | **Thickness 1** | **Thickness 2** | **Mean thickness** |
| --- | --- | --- | --- | --- | --- |
| BSY006-307 | n5 | 60.62 | 12.96 | 12.54 | 12.75 |
| TCH007-252 | n2 | 60.34 | 10.30 | 11.07 | 10.685 |
|  | n3 | 60.34 | 10.87 | 11.38 | 11.125 |
|  | n4 | 53.94 | 9.68 | 8.81 | 9.245 |
|  | n5 | 53.33 | 9.38 | 9.70 | 9.54 |
| SCR011-140 | n3 | 60.83 | 10.98 | 9.90 | 10.44 |
|  | n4 | 57.72 | 11.83 | 12.53 | 12.18 |
|  | n5 | 53.88 | 9.69 | 9.62 | 9.655 |
| BSY009-815 | n2 | 60.87 | 14.39 | 14.30 | 14.345 |
|  | n3 | 60.08 | 14.19 | 14.45 | 14.32 |
|  | n4 | 53.81 | 13.48 | 13.25 | 13.365 |
|  | n5 | 53.71 | 12.75 | 13.33 | 13.04 |
| BSY007-257 | n3 | 47.00 | 10.03 | 10.07 | 10.05 |
|  | n4 | 44.26 | 9.68 | 9.95 | 9.815 |
|  | n5 | 44.54 | 8.75 | 9.17 | 8.96 |
| SCR011-148 | n2 | 57.60 | 12.66 | 11.94 | 12.3 |
|  | n3 | 54.37 | - | 11.91 | 11.91 |
|  | n4 | 51.61 | 13.63 | 14.23 | 13.93 |
|  | n5 | 47.96 | 11.53 | 12.44 | 11.985 |
| SCR011-413 | n3 | 58.26 | 11.96 | 12.34 | 12.15 |
|  | n4 | 51.69 | 11.83 | 11.81 | 11.82 |
| TCH006-1420 | n2 | 61.70 | 13.08 | 13.20 | 13.14 |
|  | n4 | 55.60 | 11.35 | 10.94 | 11.145 |
|  | n5 | 50.96 | 10.24 | 11.11 | 10.675 |
| SCR011-276 | n3 | 65.31 | 12.65 | 12.94 | 12.795 |
|  | n4 | 55.83 | 13.55 | 13.75 | 13.65 |
| VTT006-299 | n2 | 63.19 | 12.10 | 11.63 | 11.865 |
|  | n3 | 67.34 | 12.22 | 12.11 | 12.165 |
|  | n4 | 55.85 | - | 12.49 | 12.49 |
|  | n5 | 59.17 | - | 12.16 | 12.16 |
| TCH005-16 | n2 | 65.38 | 12.87 | 12.51 | 12.69 |
|  | n3 | 63.94 | 12.26 | 12.51 | 12.385 |
|  | n4 | 57.54 | 11.42 | 11.72 | 11.57 |
|  | n5 | 57.43 | 8.68 | 10.04 | 9.36 |
| TCH005-464 | n3 | 66.89 | 11.47 | - | 11.47 |
|  | n4 | 54.19 | 12.34 | - | 12.34 |
|  | n5 | 50.34 | 11.32 | 11.24 | 11.28 |
| TCH005-21 | n2 | 61.39 | 15.35 | 14.29 | 14.82 |
|  | n3 | 67.37 | 14.29 | 13.94 | 14.115 |
|  | n5 | 60.29 | 11.13 | 9.96 | 10.545 |
| SCR011-37 | n2 | 51.25 | 10.56 | 9.95 | 10.255 |
|  | n4 | 54.54 | 12.67 | - | 12.67 |
| BSY008-206 | n2 | 63.32 | 13.24 | 13.16 | 13.2 |
|  | n4 | 55.30 | 12.66 | 12.47 | 12.565 |
| SCR010-1279 | n2 | 67.21 | 12.79 | 11.86 | 12.325 |
|  | n5 | 63.18 | 10.06 | 11.66 | 10.86 |
| VTT006-579 | n2 | 56.38 | 11.24 | 11.42 | 11.33 |
|  | n4 | 52.27 | 13.68 | 13.34 | 13.51 |
|  | n5 | 56.30 | 10.73 | 10.83 | 10.78 |
| TCH006-145 | n3 | 57.81 | 10.37 | 10.79 | 10.58 |
|  | n4 | 49.45 | 12.82 | 12.94 | 12.88 |
|  | n5 | 49.71 | 11.05 | 10.07 | 10.56 |
| BSY009-892 | n4 | 61.43 | 13.24 | 13.28 | 13.26 |
|  | n5 | 58.89 | 12.68 | 12.19 | 12.435 |
| TCH005-819 | n4 | 60.40 | 12.16 | 12.16 | 12.16 |
| BSY006-326 | n2 | 54.85 | 10.93 | 10.83 | 10.88 |
|  | n3 | 53.88 | 11.04 | 10.36 | 10.7 |
| SCR010-1009 | n3 | 62.44 | 11.16 | 10.19 | 10.675 |
|  | n4 | 53.01 | 12.28 | 12.50 | 12.39 |
|  | n5 | 54.14 | 11.00 | 10.88 | 10.94 |
| SCR011-160 | n2 | 61.31 | 11.21 | 12.21 | 11.71 |
|  | n3 | 60.87 | 12.03 | - | 12.03 |
|  | n4 | 56.97 | 13.54 | 12.70 | 13.12 |
|  | n5 | 55.80 | 12.97 | 12.01 | 12.49 |
| SCR010-1196 | n4 | 54.86 | 12.95 | 12.43 | 12.69 |
|  | n5 | 55.38 | 12.16 | 12.54 | 12.35 |
| TCH006-767 | n2 | 51.05 | 12.98 | 13.14 | 13.06 |
|  | n3 | 54.08 | 13.31 | 13.58 | 13.445 |
|  | n4 | 47.88 | 13.27 | 13.27 | 13.27 |
|  | n5 | 48.84 | 10.83 | 10.61 | 10.72 |

***Plesiochelys etalloni***

| **Specimen** | **Neural number** | **Length** | **Thickness 1** | **Thickness 2** | **Mean thickness** |
| --- | --- | --- | --- | --- | --- |
| BSY009-694 | n2 | 51.08 | 13.57 | 12.65 | 13.11 |
|  | n3 | 65.45 | 16.24 | 19.59 | 17.915 |
|  | n4 | 56.67 | 14.09 | 15.39 | 14.74 |
|  | n5 | 57.86 | 13.65 | 13.46 | 13.555 |
| SCR011-415 | n2 | 49.07 | 15.66 | 14.80 | 15.23 |
|  | n4 | 47.94 | 14.08 | 14.20 | 14.14 |
|  | n5 | 47.20 | 12.12 | 10.77 | 11.445 |
| BSY006-347 | n3 | 56.93 | 14.67 | 13.29 | 13.98 |
|  | n4 | 55.96 | 15.51 | 15.32 | 15.415 |
| BSY007-205 | n2 | 60.21 | 15.40 | 15.42 | 15.41 |
| SCR008-33 | n2 | 56.54 | 18.43 | 19.22 | 18.825 |
|  | n3 | 58.81 | 21.24 | - | 21.24 |
|  | n4 | 49.77 | - | 13.76 | 13.76 |
|  | n5 | 54.90 | 17.32 | 17.08 | 17.2 |
| SCR010-382 | n2 | 51.95 | 12.31 | 12.85 | 12.58 |
|  | n3 | 48.00 | 14.50 | 13.33 | 13.915 |
|  | n4 | 46.93 | 13.92 | 13.40 | 13.66 |
|  | n5 | 49.31 | 11.62 | 10.59 | 11.105 |
| TCH007-265 | n2 | 47.99 | 14.02 | - | 14.02 |
|  | n3 | 57.51 | 15.19 | 13.80 | 14.495 |
|  | n5 | 52.20 | 16.07 | 16.21 | 16.14 |
| TCH007-505 | n2 | 51.17 | 14.60 | 15.05 | 14.825 |
|  | n4 | 53.52 | 15.26 | 14.87 | 15.065 |
|  | n5 | 59.94 | 14.11 | 13.96 | 14.035 |

***Plesiochelys* sp.**

| **Specimen** | **Neural number** | **Length** | **Thickness 1** | **Thickness 2** | **Mean thickness** |
| --- | --- | --- | --- | --- | --- |
| BSY009-310 | n2 | 51.64 | - | 13.30 | 13.30 |
|  | n3 | 57.78 | 13.68 | 13.00 | 13.34 |
|  | n4 | 50.42 | 13.30 | 13.70 | 13.50 |
|  | n5 | 53.10 | - | 12.33 | 12.33 |
| TCH007-272 | n2 | 49.66 | 13.92 | 13.48 | 13.7 |
|  | n4 | 48.59 | 11.04 | 11.59 | 11.315 |
|  | n5 | 47.53 | 9.88 | 9.90 | 9.89 |
| TCH005-817 | n3 | 49.98 | - | 14.39 | 14.39 |
|  | n4 | 49.01 | - | 15.23 | 15.23 |
| SCR010-450 | n2 | 54.53 | 12.04 | 12.77 | 12.405 |
|  | n3 | 54.54 | 12.06 | 12.31 | 12.185 |
|  | n4 | 51.12 | 11.91 | 11.53 | 11.72 |
|  | n5 | 47.73 | 11.07 | 10.73 | 10.9 |
| SCR010-479 | n2 | 44.50 | 11.60 | 11.08 | 11.34 |
| SCR011-525 | n2 | 47.38 | 12.41 | 12.84 | 12.625 |
|  | n3 | 50.28 | 11.68 | 12.21 | 11.945 |
|  | n4 | 45.91 | 13.66 | 14.06 | 13.86 |
| BSY009-619 | n3 | 53.38 | 14.85 | 15.59 | 15.22 |
| BSY008-240 | n4 | 50.19 | 10.93 | 11.04 | 10.985 |
|  | n5 | 50.84 | 9.78 | 9.11 | 9.445 |
| SCR011-111 | n2 | 50.39 | - | 14.01 | 14.01 |
|  | n4 | 46.31 | - | 13.38 | 13.38 |
| BSY008-484 | n3 | 64.36 | 14.32 | 14.93 | 14.625 |
|  | n4 | 52.89 | - | 14.57 | 14.57 |
|  | n5 | 56.15 | - | 15.11 | 15.11 |
