# Supplementary material for: The comparative osteology of Plesiochelys bigleri n. sp., a new coastal marine turtle from the Late Jurassic of Porrentruy (Switzerland)
Source: PeerJ. 2017 Jun 28;5:e3482. doi: 10.7717/peerj.3482 (PMC5493033; doi:10.7717/peerj.3482)
Supplement: Supplementary file 2 [file peerj-05-3482-s001.pdf]

MJSN TCH007-252
